# Focus on respiration

**DOI:** 10.1093/plphys/kiad041

**Published:** 2023-01-30

**Authors:** Andrew D Hanson, A Harvey Millar, Zoran Nikoloski, Danielle A Way

**Affiliations:** Horticultural Sciences Department, University of Florida, Gainesville, FL 32611, USA; ARC Centre of Excellence in Plant Energy Biology, School of Molecular Sciences, University of Western Australia, Crawley, WA 6009, Australia; Department of Bioinformatics, Institute of Biochemistry and Biology, University of Potsdam, Potsdam 14476, Germany; Systems Biology and Mathematical Modeling, Max Planck Institute of Molecular Plant Physiology, Potsdam 14476, Germany; Research School of Biology, The Australian National University, Canberra, ACT 2601, Australia; Department of Biology, Western University, London, ON, Canada N6A 5B7; Nicholas School of the Environment, Duke University, Durham, NC 27708, USA

Respiration is as central to plant metabolism as photosynthesis; it is the main source of ATP, reducing equivalents and biosynthetic intermediates in nongreen tissues and in green tissues during the night. Further, respiration returns to the atmosphere roughly half the carbon fixed by photosynthesis at both the individual plant level (Amthor et al. 2019) and on a global scale (Raich et al. 2014) and so is a major item in the global carbon budget. In fact, terrestrial plant respiration releases about 6 times more CO_2_ to the atmosphere than the anthropogenic total from fossil fuel consumption, cement production, and land-use change (∼60 versus ∼10 gigatonnes CO_2_ per year) (NASEM 2019). Crop respiration can be reckoned to contribute around 8 gigatonnes of CO_2_ to the terrestrial total annually (Field et al. 1998; Joshi et al. 2023), making crop CO_2_ emissions alone almost as high as anthropogenic emissions.

**Figure 1.**
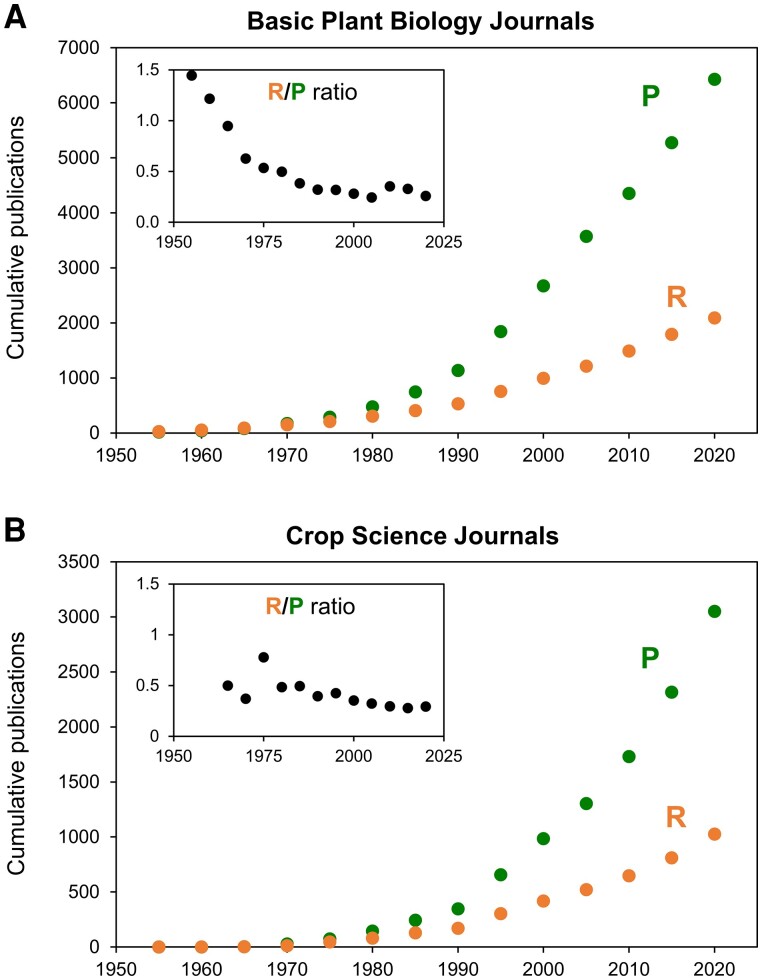
Cumulative number of publications on photosynthesis (P) and respiration (R) between 1950 and 2020. **A)** Publications in representative basic plant biology journals (i.e. *Plant Physiology*, *Plant Cell*, *New Phytologist*, *Plant Journal*, *Plant Cell and Environment*, *Molecular Plant*, *Nature Plants*, *Plant Biology*, *Trends in Plant Science*, *Annual Review of Plant Biology*, or *Annual Review of Plant Physiology* and *Plant Molecular Biology*, *Annual Review of Plant Physiology*). **B)** Publications in representative crop science journals (i.e. *Field Crops Research*, *Advances in Agronomy*, *Agronomy Journal*, *Journal of Agricultural Science*, *European Journal of Agronomy*, *Crop Science*, *Canadian Journal of Plant Science*, *Australian Journal of Crop Science*, *Journal of the American Society for Horticultural Science*, *HortScience*, *Horticulture Research*, *Scientia Horticulturae*). The insets in each plot show the ratio of respiration to photosynthesis publications (R/P). Data were extracted from the Web of Science database using either “photosynthesis” or “respiration + plant” as search terms along with the list of journal names.

Despite the cardinal metabolic and biogeochemical significance of plant respiration and the potential to increase crop yields by cutting respiratory CO_2_ loss (Amthor et al. 2019; Garcia et al. 2023; Joshi et al. 2023), plant respiration has long had far less research attention than photosynthesis—a situation that has been justly called an “asymmetry in crop-focused academic research” (Reynolds et al. 2021). This asymmetry is readily apparent from a search of plant science journals for articles relating to photosynthesis or respiration over the past 70 yr (Fig. 1). The bias in favor of photosynthesis is currently about 3-fold in the major journals that cover both “basic plant biology” (Fig. 1A) and “crop science” (Fig. 1B) (a somewhat arbitrary but still useful division). This bias has apparently increased over time, more markedly in basic science journals (which emphasize molecular mechanisms) (Fig. 1A, inset) than in crop science journals (which focus on physiology, environmental responses, and breeding) (Fig. 1B, inset). It would thus seem that our grandparents—particularly reductionist ones—were more interested in understanding respiration than we have been in recent decades.

This situation is ultimately unreasonable and a more symmetrical research investment in respiration will benefit plant science as a whole. This Focus Issue was inspired by the need to take stock of the progress now being made and to encourage further progress. It adds to recent reviews overviewing the progress in research on respiration rates (Dusenge et al. 2019; O’Leary et al. 2019), plant mitochondrial composition and activity (Møller et al. 2021), and assembly and function of the plant respiratory oxidative phosphorylation system (Meyer et al. 2019, 2022). It also complements the seminal review of Cannel and Thornley (2000) on guiding principles for modeling plant respiration. To these ends, the issue includes a set of Update Reviews that span the range of respiration research from the plant community level to subcellular and enzyme levels, as well as Research Articles.

Arranged roughly in order of descending organizational level, the Updates are as follows. Bulut et al. (2023) survey the (still scant) literature on natural variation in plant respiration and the (more extensive) literature on variation in respiratory metabolites and other respiration-related traits. They end their review with a clarion call for more work on natural variation of respiration itself and on its mechanistic and evolutionary basis. Relatedly, O’Leary et al. (2023) review substantial advancements in measuring plant respiration, particularly in high-throughput modes compatible with large-scale ecological surveys, genetic screens, crop breeding trials, and omics studies. They point to a future in which it will be possible to link respiratory variation to specific genes, benefitting basic knowledge as well as crop improvement. Wendering and Nikoloski (2023) cover advancements in computational modeling of plant respiration, particularly the increasing use of mechanistic (i.e. biochemically based) models. They emphasize the potential value of such models to metabolic engineering of, and breeding for, a particular (average) respiration rate and their use in terrestrial biosphere models that simulate responses to climate change.

Bridging between the crop level and biochemical processes, Bathe et al. (2023) revisit the principles of plant respiratory energy budgeting and estimating the costs of metabolic processes. They then apply these principles to assess how synthetic biology interventions that add new metabolic demands or cut existing ones could affect crop yield and carbon sequestration. Moving to the organelle level, Igamberdiev and Bykova (2023) summarize the advancements in understanding the unique roles of mitochondrial metabolism in photosynthetic tissues in light, including the operation of tricarboxylic acid cycle enzymes in noncyclic mode and noncoupled electron transport. Overall, they present an emerging new view of how mitochondria in photosynthetic cells transition from powerhouses to thermodynamic buffering units that regulate cellular redox and energy balance and furnish intermediates and reducing power to support biosynthesis. At the level of the whole respiratory chain, Ghifari et al. (2023) discuss advancements in understanding the makeup, biogenesis, and turnover of plant oxidative phosphorylation complexes and mechanisms that regulate their biogenesis and activity. At the individual enzyme level, McDonald (2023) covers advancements in defining the distribution and physiological functions of the alternative oxidase of the plant mitochondrial electron transport chain and anticipates the progress that genome editing tools and new oxygen sensing technologies now make possible. Finally, at the metabolite level, Le and Millar (2023) cover the diverse respiratory substrates that mitochondria import and use in normal and stress conditions, highlighting evidence for metabolic channeling in supply of substrates to respiration. With this as a base, they then consider how synthetic biology could engineer bypasses to allow the use of alternative respiratory substrates, with potential benefits for carbon-use efficiency and growth.

Reinforcing the disparity between the number of research publications on respiration and photosynthesis in Fig. 1, this issue's Updates are shot through with phrases such as “neglected historically,” “paucity of studies,” “relatively rare,” “poorly known,” “still elementary,” “always had far less attention,” and “attracted considerably less…efforts in comparison to photosynthesis.” It is interesting to note that achievements in photosynthesis research have been greatly aided by a community-wide adoption of grand challenges surrounding the wavelengths of light used by light-harvesting machinery, re-engineering Rubisco, building bypasses to photorespiration, and introducing C_4_ photosynthesis into C_3_ crops. Plant respiration research has not yet rallied around such grand challenges and this may continue to limit its further progress in a global era of science as an enabler of change, not just a tool of discovery. Another major reason for the advancements in photosynthesis research is the accumulated knowledge about the few canonical pathways underlying photosynthesis, allowing the development of descriptive models (e.g. Farquhar et al. 1980) and guiding the design of improvement strategies. In contrast, as the Updates remind us, measuring, characterizing, and engineering respiration require a systems perspective, whose potential is not fully exploited despite advancements in plant systems biology. These Updates seek to spark this process, in confident expectation of a bright future for knowledge of plant respiration and its application to agricultural productivity and climate change mitigation.
